# Bayesian Phylogeography Finds Its Roots

**DOI:** 10.1371/journal.pcbi.1000520

**Published:** 2009-09-25

**Authors:** Philippe Lemey, Andrew Rambaut, Alexei J. Drummond, Marc A. Suchard

**Affiliations:** 1 Department of Microbiology and Immunology, Katholieke Universiteit Leuven, Leuven, Belgium; 2 Institute of Evolutionary Biology, University of Edinburgh, Edinburgh, United Kingdom; 3 Department of Computer Science, University of Auckland, Auckland, New Zealand; 4 Departments of Biomathematics and Human Genetics, David Geffen School of Medicine, University of California, Los Angeles, California, United States of America; 5 Department of Biostatistics, School of Public Health, University of California, Los Angeles, California, United States of America; Imperial College London, United Kingdom

## Abstract

As a key factor in endemic and epidemic dynamics, the geographical distribution of viruses has been frequently interpreted in the light of their genetic histories. Unfortunately, inference of historical dispersal or migration patterns of viruses has mainly been restricted to model-free heuristic approaches that provide little insight into the temporal setting of the spatial dynamics. The introduction of probabilistic models of evolution, however, offers unique opportunities to engage in this statistical endeavor. Here we introduce a Bayesian framework for inference, visualization and hypothesis testing of phylogeographic history. By implementing character mapping in a Bayesian software that samples time-scaled phylogenies, we enable the reconstruction of timed viral dispersal patterns while accommodating phylogenetic uncertainty. Standard Markov model inference is extended with a stochastic search variable selection procedure that identifies the parsimonious descriptions of the diffusion process. In addition, we propose priors that can incorporate geographical sampling distributions or characterize alternative hypotheses about the spatial dynamics. To visualize the spatial and temporal information, we summarize inferences using virtual globe software. We describe how Bayesian phylogeography compares with previous parsimony analysis in the investigation of the influenza A H5N1 origin and H5N1 epidemiological linkage among sampling localities. Analysis of rabies in West African dog populations reveals how virus diffusion may enable endemic maintenance through continuous epidemic cycles. From these analyses, we conclude that our phylogeographic framework will make an important asset in molecular epidemiology that can be easily generalized to infer biogeogeography from genetic data for many organisms.

## Author Summary

Spreading in time and space, rapidly evolving viruses can accumulate a considerable amount of genetic variation. As a consequence, viral genomes become valuable resources to reconstruct the spatial and temporal processes that are shaping epidemic or endemic dynamics. In molecular epidemiology, spatial inference is often limited to the interpretation of evolutionary histories with respect to the sampling locations of the pathogens. To test hypotheses about the spatial diffusion patterns of viruses, analytical techniques are required that enable us to reconstruct how viruses migrated in the past. Here, we develop a model to infer diffusion processes among discrete locations in timed evolutionary histories in a statistically efficient fashion. Applications to Avian Influenza A H5N1 and Rabies virus in Central and West African dogs demonstrate several advantages of simultaneously inferring spatial and temporal processes from gene sequences.

## Introduction

Phylogenetic inference from molecular sequences is becoming an increasingly popular tool to trace the patterns of pathogen dispersal. The time-scale of epidemic spread usually provides ample time for rapidly evolving viruses to accumulate informative mutations in their genomes [1]. As a consequence, spatial diffusion—among other processes—can leave a measurable footprint in sampled gene sequences from these viruses [1]. Reconstructing both the evolutionary history and spatial process from these sequences provides fundamental understanding of the evolutionary dynamics underlying epidemics, e.g. [2],[3]. It is also hoped that these insights can be translated to effective intervention and prevention strategies [4] and elucidating the key factors in viral transmission and gene flow over larger distances is central in formulating such strategies, e.g. [5].

Phylogeographic analyses are a common approach in molecular ecology, connecting historical processes in evolution with spatial distributions that traditionally scale over millions of years [6]. Many popular phylogeographic approaches [7],[8] can be remiss in ignoring the interaction between evolutionary processes and spatial-temporal domains. One first reconstructs a phylogeny omitting spatial information and then conditions the phylogeographic inferences on this reconstruction [1],[9], exploiting non-parametric tests to evaluate the significance of this conditional structure, e.g. [7],[10],[11]. To draw conclusions about the epidemic origin or epidemiological linkage between locations, however, we require a reconstruction of the dispersal patterns and process throughout the evolutionary history. Considering locations as discrete states, this boils down to the well-known problem of ancestral state inference [7]. Parsimony is a popular heuristic approach to map characters onto a single phylogenetic tree [12]. Unfortunately, parsimony reconstructions ignore important sources of model uncertainty, including both uncertainty in the dispersal process as well as in the unknown phylogeny [13]. In addition, minimizing the number of state exchanges over a phylogeny is misleading when rates of evolution are rapid and when the state exchange probabilities are unequal [14].

Probabilistic methods draw on an explicit model of state evolution, permitting the ability to glimpse the complete state history over the entire phylogeny and conveniently draw statistical inferences [15]–[17]. These analyses typically employ continuous-time Markov chain models for discrete state evolution analogous to common nucleotide, codon or amino acid substitution models [18]. In contrast to parsimony, maximum likelihood-based reconstructions incorporate branch length differences in calculating the conditional probability of each ancestral state given the observed states at the phylogeny tips [14]. Bayesian reconstruction methods enable further generalization of this conditional probability analysis by removing the necessity to fix the Markov model parameters to obtain ancestral states and the necessity to specify a fixed tree topology with known branch lengths. Bayesian inference integrates conclusions over all possible parameter values but to achieve this, however, requires prior probability distributions for all aspects of the model.

While probabilistic methods have been previously presented in a bio- or phylogeographic context, in particular Bayesian methods that integrate over phylogenetic uncertainty and Markov model parameter uncertainty [19], viral phylogeography studies have rarely made use of these developments. This may be a consequence of low awareness of existing software implementations for arbitrary continuous-time Markov chain models [20],[21] or a lack of appreciation for the uncertainty intrinsic in these reconstructions and the ease with which one can formally access epidemiological linkage through probabilistic approaches. A recent phylogeographic study of influenza A H5N1 introduces a heuristic non-parametric test to evaluate whether parsimony-inferred migration events between two particular locations occur at significantly high frequency [22]. Null distributions for these frequencies arise from randomizing tip localities after false discovery rate correction to control for simultaneous testing issues. Although this procedure addresses concerns about statistical inference on sparse frequency matrices, the multiple comparison correction still results in a conservative estimate of significant migration events. Fully probabilistic approaches may further ease statistical inference, yet similar tests remain lacking for likelihood-based phylogeographic models.

Advances in evolutionary inference methodology have frequently demonstrated how novel approaches can be appended to a sequence of analyses, in many cases starting from alignment to parameter estimation conditional on tree reconstructions. For example, demographic inference has involved genealogy reconstruction, estimating a time scale for the evolutionary history, and coalescent theory to quantify the demographic impact on this tree shape [23]. It is well acknowledged that such sequential procedures ignore important sources of uncertainty because they generally purge error associated with each intermediate estimate. With the advent of novel computational techniques like Markov chain Monte Carlo (MCMC) sampling, it has become feasible to integrate many of the models involved and simultaneously estimate parameters of interest. Demographic inference is a well-known example of genealogy-based population genetics that benefited from these advances [24],[25]. Bayesian MCMC methods also enable ancestral state reconstruction while simultaneously accounting for both phylogenetic and mapping uncertainty. Although this adds much needed credibility to ancestral reconstruction [13], phylogeographic analysis would benefit even more from fully integrating spatial, temporal and demographic inference.

Here, we implement ancestral reconstruction of discrete states in a Bayesian statistical framework for evolutionary hypothesis testing that is geared towards rooted, time-measured phylogenies. This allows character mapping in natural time scales, calibrated under a strict or relaxed molecular clock, in combination with several models of population size change. We use this full probabilistic approach to study viral phylogeography and extend the Bayesian implementation to a mixture model in which exchange rates in the Markov model are allowed to be zero with some probability. This Bayesian stochastic search variable selection (BSSVS) enables us to construct a Bayes factor test that identifies the most parsimonious description of the phylogeographic diffusion process. We also demonstrate how the geographical distribution of the sampling locations can be incorporated as prior specifications. Through feature-rich visual summaries of the space-time process, we demonstrate how this approach can offer insights into the spatial epidemic history of Avian influenza A-H5N1 and rabies viruses in Africa.

The highly pathogenic avian influenza A-H5N1 viruses have been present for over a decade in Southern China and spread in multiple waves to different types of poultry in countries across Asia, Africa and Europe [26]. As a result, highly pathogenic A-H5N1 is now a panzootic disease and represents a continuous threat for human spill-over. Strong surveillance has been in place since these viruses caused extensive outbreaks, but the source and early dissemination pathways have remained uncertain. Because parsimony analysis has attempted to shed light on the latter [22], A-H5N1 provides an ideal example for comparison with Bayesian phylogeographic inference. Rabies is endemic in Asia and Africa, where the primary reservoir and vector for rabies virus (RABV) is the domestic dog. Phylogenetic analysis has revealed several genotypes of lyssaviruses (family *Rhabdoviridae*); genotype 1 has been found responsible for classical rabies, a fatal disease in terrestrial mammals throughout the world [27],[28]. Here, we explore the phylogeographic history of RABV in domestic dogs in West Central Africa, using recently obtained sequence data, and evaluate the role of viral dispersal in maintaining RABV epidemic cycles.

## Results

We examine the evolution and spatial dispersion of two viral pathogens, Avian influenza A-H5N1 and rabies, to demonstrate the strengths and limitations of our discretized stochastic model for phylogeography.

### Avian influenza A-H5N1

To reconstruct the spatial dispersion patterns of Avian influenza A-H5N1, we analyze the hemagglutinin (HA) and neuraminidase (NA) gene datasets previously compiled by [22]. Both datasets contain whole gene sequences from 192 A-H5N1 strains sampled from 20 localities across Eurasia. [22] explore these genes individually, as well as concatenated together, through a strictly parsimony-based ancestral reconstruction method. Our Bayesian approach builds upon stochastic models and naturally affords quantification of uncertainty in both the ancestral state reconstructions and the underlying phylogeographic process. Further, as we are able to infer plausible root positions unlike the original analysis, we are not required to include outgroup sequences. To model sequence evolution, we employ the [29] (HKY85) CTMC model of nucleotide substitution; we include discrete gamma-distributed rate variation [30] and assume an unknown, constant population-size coalescent process prior over the unknown phylogeny [31]. Exploratory analyses using the less restrictive Bayesian skyline plot model indicate that the demographic prior has little influence on the phylogeographic inference (data not shown).

**Figure 1:**
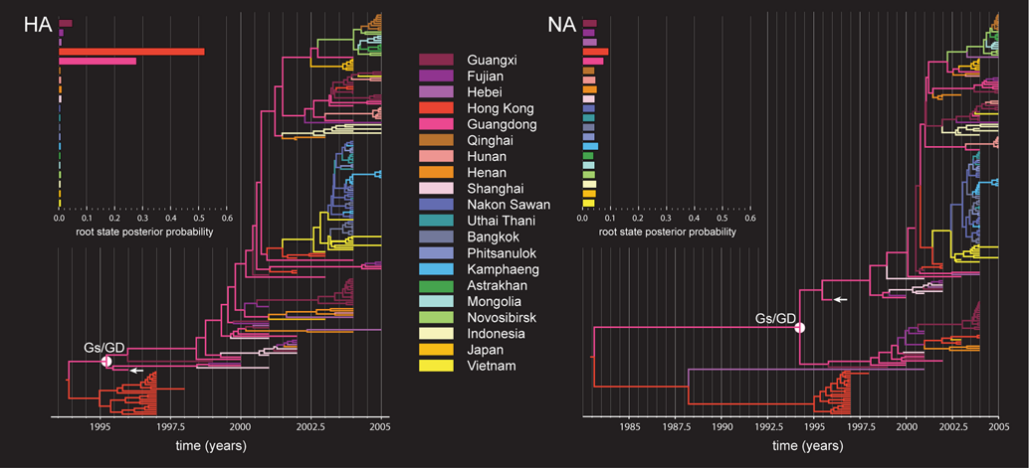
Maximum clade credibility (MCC) phylogenies for hemagglutinin (HA) and neuraminidase (NA) genes of Avian influenza A-H5N1. We color branches according to the most probable location state of their descendent nodes. We use the same color coding as [22]. To the upper left of both phylogenies are their root location state posterior probability distributions. A white arrow indicates the A/goose/Guangdong/1/96 sequence; a filled white circle identifies the most recent common ancestor of the Gs/GD lineage named after this strain.

Figure 1 summarizes the Bayesian maximum clade credibility (MCC) trees for the A-H5N1 HA and NA segments. An MCC tree is a point-estimate characterizing the posterior distribution of trees and represents the tree topology yielding the highest product of individual clade probabilities in their posterior sample [2]; branch lengths in these MCC trees are posterior median estimates.

We further annotated the tree nodes with their most probable (modal) location states via color labelings. Although the nucleotide substitution rates are very similar across genes (HA: posterior mean 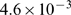, 95% Bayesian credible interval 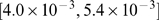; NA: 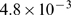 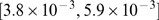), the root lies considerably deeper in the NA tree resulting in a time scale that spans about twice the time of the HA tree. In combination with other topological differences between the trees, this difference strongly suggests past reassortment events between both segments, with the progenitor virus of the basal Hong Kong clade and a chicken strain from Hebei having acquired an NA segment from different lineages. Such events are not surprising given frequent reports of A-H5N1 reassortment in China, e.g. [26], and the particular reassortment event for the basal Hong Kong clade has very recently been confirmed [32].

Despite different time scales for HA and NA, most probable location states agree on Guangdong as the predominant location of these sequences throughout the majority of their evolutionary history. As an indication of the A-H5N1 epidemic origin, we consider the inferred location at the root of the trees (Figure 1). In the HA tree, Guangdong and Hong Kong share a vast majority of the posterior mass, neighboring locations in which surveillance efforts report early Avian influenza cases [33],[34]. In the NA tree, although Hong Kong and Guangdong still obtain marginally higher support than other locations, all posterior root state probabilities are much closer to their prior probability. The substantially deeper NA root explains this difference as the depth greatly increases uncertainty on the root state. Table 1 quantifies differences in ancestral state reconstruction uncertainty between the HA and NA trees using the Kullback-Leibler (KL) divergence measure (see Methods). The NA tree results in considerably lower KL divergence than the HA tree, signifying a much smaller deviation of the posterior distribution of the root location from the prior. However, lack of phylogeographic structure in the data does not contribute to this difference because the NA trees return a lower association index (AI). This measure of spatial admixture is based on a sum across all nodes in the tree of the complement of the frequency of the most abundant location among all descendent taxa weighted by the depth of the node in the tree [35], and thus bears some relationship with an entropy value for descendent taxa locations. The AI rescales this sum by its expectation for randomized location assignments and results in low values for relatively strong phylogeny-locality correlation whereas AI values close to one reflect complete spatial admixture. If the basal Hong Kong clade and a chicken strain from Hebei have indeed acquired a different NA through reassortment, the root state might be difficult to interpret for NA and is not necessarily the same as that for HA. Therefore, we also list uncertainty measures for the marginal posterior distribution of the most recent common ancestor (MRCA) of the Gs/GD lineage, named after the A/goose/Guangdong/1/96 strain very close to this node (indicated in Figure 1). KL divergence is again lower for this node in the NA phylogeny, but the difference is not as pronounced as for the root node.

**Table 1:** Mapping uncertainty and model exploration for Avian influenza A-H5N1 hemagglutinin (HA) and neuroaminidase (NA) genes.

Data	Model	Kullback-Leibler		Association index
		root	GsGD	
HA	C	1.4464	2.1999	0.21 (0.17–0.25)
NA	C	0.0184	1.6679	0.14 (0.09–0.18)
HA	C, BSSVS	1.7895	1.4383	0.24 (0.19–0.29)
NA	C, BSSVS	0.5660	1.1185	0.20 (0.14–0.26)
HA	D, BSSVS	1.7861	1.4059	0.25 (0.20–0.30)
NA	D, BSSVS	0.5811	1.1889	0.23 (0.17–0.29)
Shared	C	HA: 1.4704	HA: 2.2303	HA: 0.21 (0.17–0.25)
		NA: 0.0321	NA: 1.7281	NA: 0.15 (0.10–0.19)
Shared	C, BSSVS	HA: 1.8965	HA: 1.5844	HA: 0.25 (0.21–0.30)
		NA: 0.7813	NA: 1.2511	NA: 0.22 (0.16–0.28)
Shared	DI, BSSVS	HA: 1.8038	HA: 1.6086	HA: 0.26 (0.21–0.31)
		NA: 0.7748	NA: 1.3195	NA: 0.23 (0.17–0.29)
HA (fixed)	C	1.5030	2.5626	0.18
HA (fixed)	C, BSSVS	1.7578	1.7026	0.18
HA (fixed)	DI, BSSVS	1.7235	1.7364	0.18

We report the Kullback-Leibler divergence between the posterior and prior location distributions of the root and the GsGD most recent common ancestor (MRCA), as well as a phylogeographic association index. We analyze genes independently, assuming equal phylogeographic models (Shared) and by fixing the HA phylogeny through phylogeographic models with prior rates proportional to a constant (C) or distance-informed (DI) and using Bayesian stochastic search variable selection (BSSVS).

Table 1 also explores the effects of distance-informed priors and BSSVS on location reconstruction. In general, the distance-informed priors furnish little advantage while inferring the root locations for both the HA and NA trees. If anything, KL divergences are slightly smaller for models involving distance-informed priors than those with flat priors. For these data, this finding is unsurprising as physical distances can be poor proxies for inverse-diffusion rates when dispersal results from a heterogenous mix of migratory birds, transport of poultry and poultry products, and trade of wild birds [36]. Finally, we also investigated the uncertainty that is accommodated by averaging over plausible trees by analyzing the HA data using a fixed tree topology and branch lengths (Table 1). The state reconstructions for the Gs/GD node in the fixed tree topology appear to ignore some uncertainty in comparison to integrating trees, which is not that evident for the root node. Although state reconstruction uncertainty is expected to be correlated among nodes, we also compared the KL divergence summed over all internal nodes, indicating much higher KL divergences using a fixed tree topology, e.g. for HA, 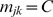: 292 vs 523 for integrating trees and a fixed tree respectively.

**Figure 2:**
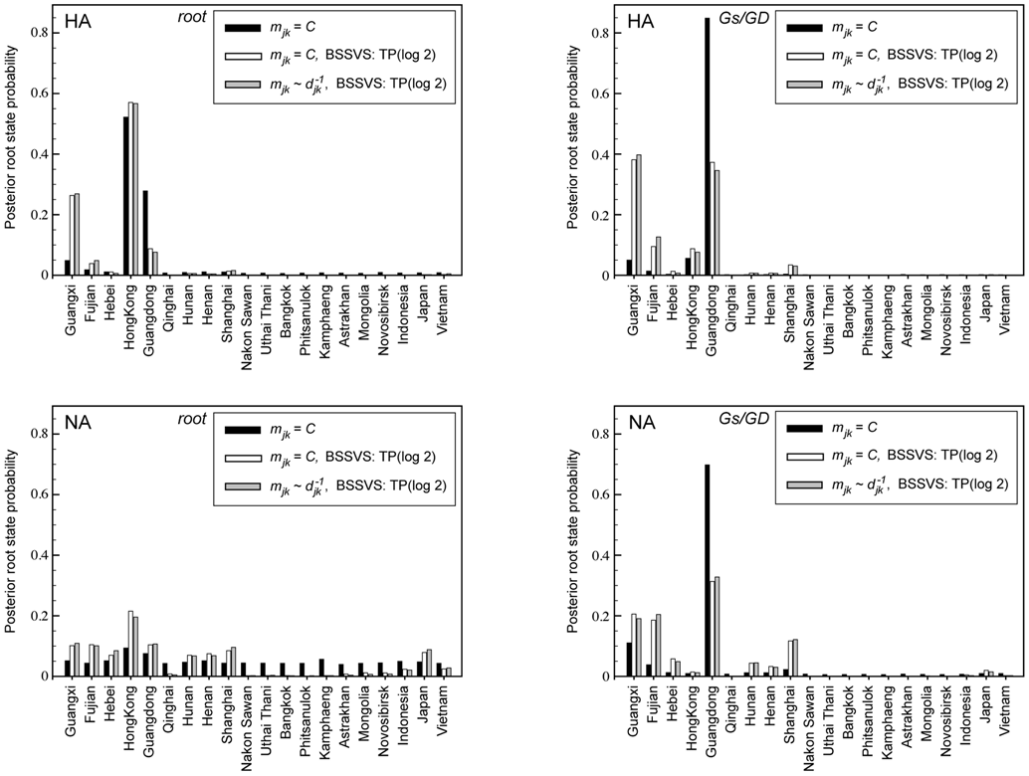
Posterior location probabilities at two different nodes, the root and GsGD MRCA, for different Bayesian phylogeographic analyses of Avian influenza A-H5N1 HA and NA. The posterior probabilities are shown for different expectations, 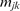, for the gamma priors on the rates; either 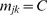, where 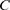 is an arbitrary constant, or 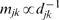, where 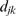 is the distance between location 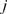 and 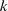. A truncated Poisson (TP) prior with 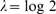 was used in the Bayesian stochastic search variable selection (BSSVS) procedure.

Under BSSVS, we assume a truncated Poisson prior that assigns 50% prior probability on the minimal rate configuration, comprising 19 non-zero rates connecting the 20 locations. This model strongly favors reduced parameterizations. A sensitivity analysis with respect to larger Poisson prior means reinforces that the data prefer a minimal number of rates, as increasing the mean leads to lower overall marginal likelihoods (Table 2). BSSVS has a strong impact on root location reconstruction (Figure 2). Many localities that are weakly supported as the root location without BSSVS obtain negligible posterior probability under BSSVS. Consequentially, BSSVS leads to larger KL divergences for both the HA and NA root nodes (Table 1), suggesting that these reduced models more efficiently exploit the information content of the data. Interestingly, the posterior support for Guangxi increases under BSSVS at the expense of Guangdong in the HA phylogeny (Figure 2). This may be an artifact of the reversible CTMC assumption we enforce. Specifically, at the tips of the phylogeny, several pathways of migration into Guangxi are highly likely. Assuming reversibility dictates that migration out of this location occurs as well; placing these emigrations deeper in the phylogeny is most consistent with the data. Because many locations already receive very low posterior probabilities at the GsGD node, the increase in posterior probability for a few locations now seems to outweigh the marginal reductions in posterior probabilities for most other locations and results in lower KL divergences at this node.

**Table 2:** Sensitivity analysis to the expected number of migration rates for A-H5N1.

Prior Mean	ML (stdev)	Posterior median (BCIs)	KL divergence
			root	Gs/GD
log(2)	−11339.343 (0.856)	21 (19–22)	1.7895	1.4383
1	−11339.670 (0.636)	21 (19–23)	1.7991	1.4540
5	−11341.197 (0.955)	25 (22–29)	1.7804	1.4533
10	−11342.463 (0.883)	29 (24–34)	1.7940	1.7940
20	−11343.429 (0.957)	36 (29–43)	1.7691	1.5691

We report estimates of 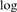 marginal likelihoods (ML) with Monte Carlo error standard deviations, posterior medians and 95% Bayesian credible intervals (BCIs) of the inferred number of rates and KL divergences across a range of prior expectations.

**Figure 3:**
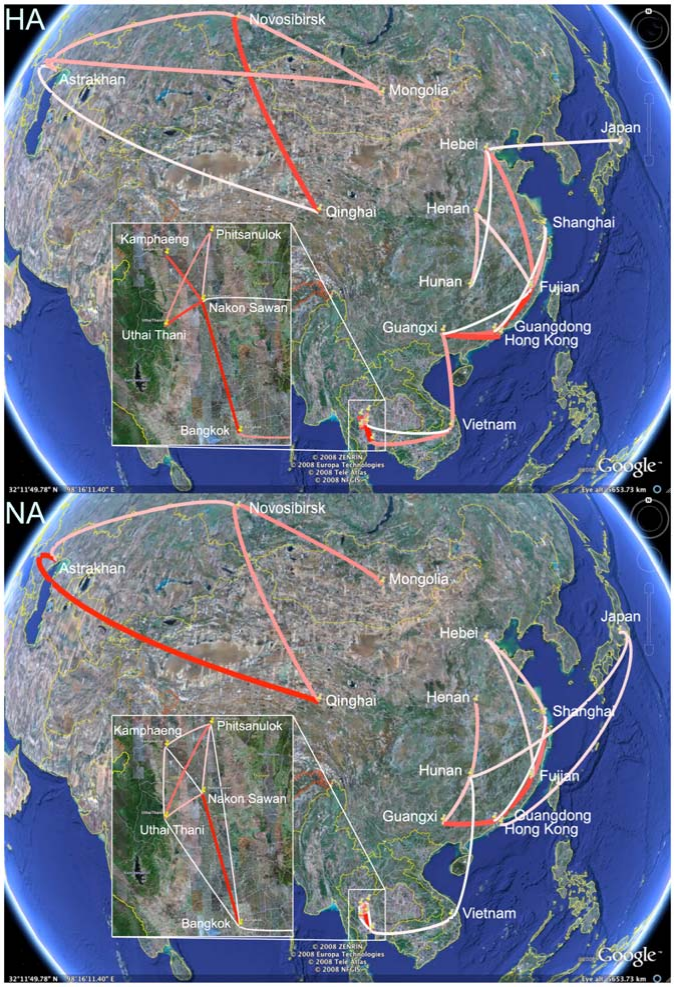
Bayes factor (BF) test for significant non-zero rates in Avian influenza A-H5N1. Only rates supported by a BF greater than 3 are indicated. The color and thickness of the line represent the relative strength by which the rates are supported; thin white lines and thick red lines suggest relatively weak and strong support respectively. The maps are based on satellite pictures made available in Google Earth (http://earth.google.com).

By specifying a prior on the number of non-zero rates, we are able to construct Bayes factor (BF) tests for significance of individual rates (Figure 3). To visualize the epidemiological linkage that this test establishes, we employ Google Earth to display all rates with a non-zero expectancy that results in a BF larger than three. The majority of well-supported rates (16 out of 25 for both genes) are concordant between HA and NA. Some variation in support for different migration pathways between HA and NA was also noted in the original parsimony analysis [22]. Importantly, Guangdong presents as an end-point in three well-supported epidemiological links in HA as well in NA. For HA, four migration links previously identified using the parsimony sFDR test (Guangdong to Fujian, Bangkok to Vietnam, Uthai Thani to Phitsanulok, and Qinghai to Novosibirsk) are also present in our well-supported symmetric rates. We can, however, not confirm epidemiological linkage directly between Guangdong and Indonesia. Despite having more supported rates by this Bayes factor test as compared to the parsimony sFDR test, it remains difficult to univocally identify the pathways seeding remote localities as Japan and Indonesia, and to connect the eastern diffusion network with the Chinese/Russian inlands. Distance-informed priors do not have strong influence on the Bayes factor test for significant rates.

**Figure 4:**
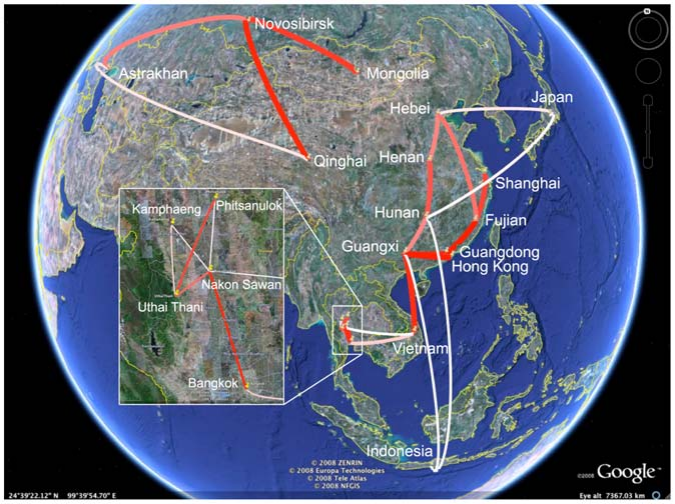
Bayes factor test for significant non-zero rates for the combined HA and NA analysis. Only rates supported by a BF greater than 3 are indicated. The color and thickness of the line represent the relative strength by which the rates are supported; thin white lines and thick red lines suggest relatively weak and strong support respectively. The maps are based on satellite pictures made available in Google Earth (http://earth.google.com).

The presence of reassortment amongst the gene segments obfuscates phylogenetic inference for concatenated HA/NA sequence data. In this respect, it is interesting to note that previous parsimony reconstructions on a phylogeny for the concatenated HA and NA segments result in fewer significant diffusion rates compared to the separate analyses; [22] found 2 for the concatenated alignment vs. 5 and 10 for HA and NA separately. The Bayesian framework enables a flexible combination of the data without having to specify a single phylogeny for both segments. To this end, we share the instantaneous rate matrix 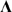 between both segment phylogenies and sample all parameters in a single MCMC analysis. Without BSSVS, sharing the rate matrix results in slightly higher KL divergences for both the root node and the Gs/GD node in the HA and NA phylogenies (Table 1). Figure 4 illustrates the well-supported rates based on the Bayes factor test of the shared rate matrix with a distance-informed prior. The shared data bring to light two possible pathways seeding the remote localities of Japan and Indonesia; these pathways suggest Guangxi and Hunan as possible source for Indonesia, and Hunan and Hebei as possible source for Japan.

**Figure 5:**
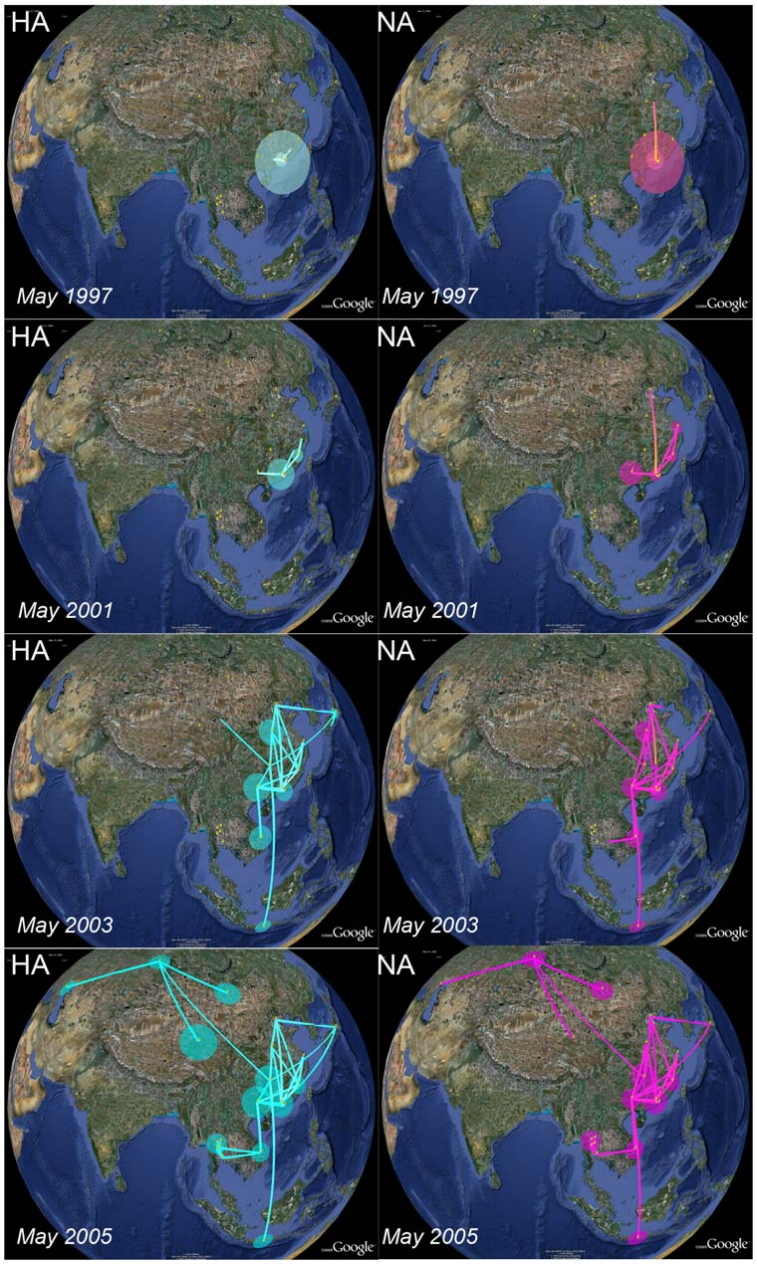
Temporal dynamics of spatial Avian influenza A-H5N1 diffusion. We provide snapshots of the dispersal pattern for May 1997, 2001, 2003 and 2005. Lines between locations represent branches in the MCC tree along which the relevant location transition occurs. Location circle diameters are proportional to square root of the number of MCC branches maintaining a particular location state at each time-point. The white-green and yellow-magenta color gradients inform the relative age of the transitions for HA and NA respectively (older-recent). The maps are based on satellite pictures made available in Google Earth (http://earth.google.com).

A major advantage of the current phylogeography implementation is the ability to infer the migration process in natural time scales. The panels in Figure 5 summarize the temporal dynamics of A-H5N1 spatial diffusion inferred using the shared rate matrix (KML files, Dataset S1 and S2 for HA and NA respectively, which enable visualizing the spread over time in Google Earth are available as supporting information). The lines connecting different locations represent branches in the MCC tree on which state exchanges occur and circle areas reflect the number of branches maintaining a particular state at that time point. By May 1997, Avian influenza lineages have accumulated in Guangdong, where the virus was originally isolated from a farmed goose [33], and to a large extent in Hong Kong (both circles overlap in the figure), where 18 cases of human infection occurred in 1997. Although significant poultry culling efforts have been made in Hong Kong, the virus continues circulating in Southern China. By May 2001, the virus appears to have spread to Guangxi, Fujian, Shanghai and Hebei in the north of China. The diffusion process intensifies by May 2003; the virus reaches more remote locations like Japan, Vietnam and Indonesia. This is known as ‘wave 1’ in Southeast Asia resulting in severe A-H5N1 outbreaks in 2003. Finally, A-H5N1 virus also spreads to the west in a second major transmission wave. Since this occurs after a major outbreak in migratory waterfowl at Qinghai Lake in Northern China, migratory birds could play a prominent role in this dissemination pathway [37].

### Rabies in dogs in West and Central Africa

We investigate the “Africa 2” lineage of rabies transmitted by African dogs. This lineage forms one of the most divergent African rabies virus clades [28],[38]. The data set we analyze here comprises 101 complete nucleoprotein (N) gene sequences sampled across 12 African countries including Chad, Niger, Cameroon, the Central African Republic, Benin, Sierra Leone, Mali, Mauritania, Guinea, Ivory Coast and Burkina Faso [39].

**Figure 6:**
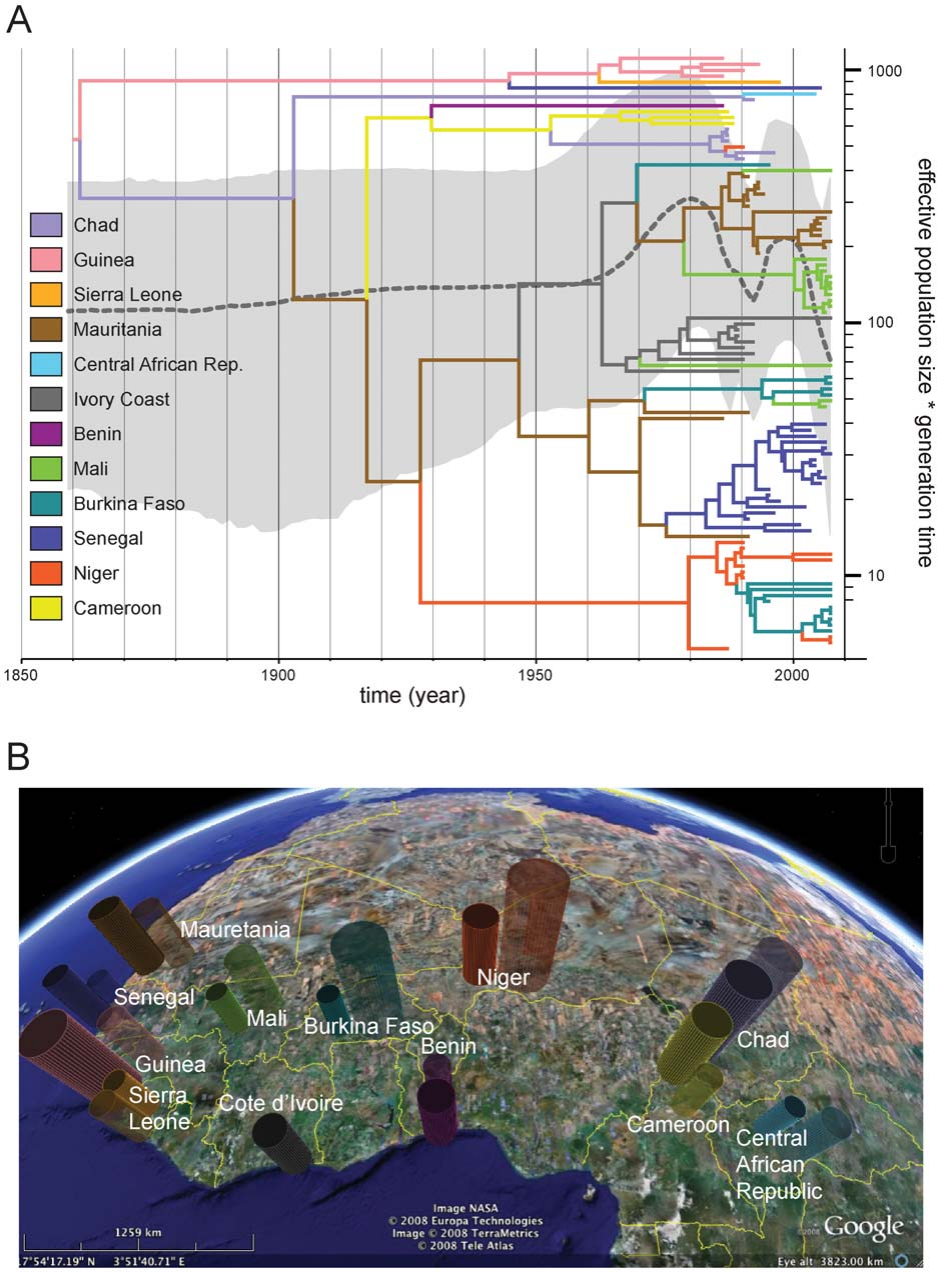
Inferred phylogeny, demographic history and root location for Africa rabies virus. (A) MCC phylogeny with branches colored according to the most probable posterior location of their child nodes; superimposed under the phylogeny lies the inferred demographic history. (B) Root location posterior probabilities are shown for the standard discrete model (opaque) and for the BSSVS extension with, in addition, distance-informed priors on the infinitesimal migration rates (transparent). The distance-informed priors in the latter had little impact on the results (data not shown). Both the height and width of the cylinders are proportional to root location posterior probability; the same colors as the tree branches in (A) are used. The maps are based on satellite pictures made available in Google Earth (http://earth.google.com.).

Figure 6A illustrates the location-annotated MCC phylogeny and demographic history for the African dog rabies lineages. We make this initial inference without either BSSVS or a distance-informed prior. To allow for variation in the underlying coalescent process giving rise to the phylogeny, we assume a piece-wise constant multiple change-point model on the effective population-size with 20 coalecent-interval groups [25]. As generally observed for rabies viruses [28], there exists strong signal of phylogenetic clustering according to sampling location. This observation is also reflected in a low AI (0.087 [0.043–0.132]). In contrast to the influenza phylogenies, however, there is no single location for which sampled sequences are phylogenetically dispersed throughout the whole phylogeny. Together with the relative deep time scale of the phylogeny and the absence of sequences sampled closer to the root, this hampers precise inference of the location state at the root. The root state posterior probabilities for all locations range between 0.059 and 0.125, with Chad and Guinea receiving the highest probability (0.115 and 0.124 respectively). These two locations are geographically distant from each other, but they both host viruses from the most basal lineage in the phylogeny (Figure 6A). Root inference is somewhat different using BSSVS and a distance-informed prior on the rates (Figure 6B). In this case, a more central location, Niger, obtains the highest posterior probability (0.144) but the KL divergence for the root state reconstruction is only marginally greater than zero (0.0645). The exploitation of BSSVS contributes to this effect; as for Avian influenza A-H5N1, distance-informed priors, alone, on the rates have little impact (data not shown).

**Figure 7:**
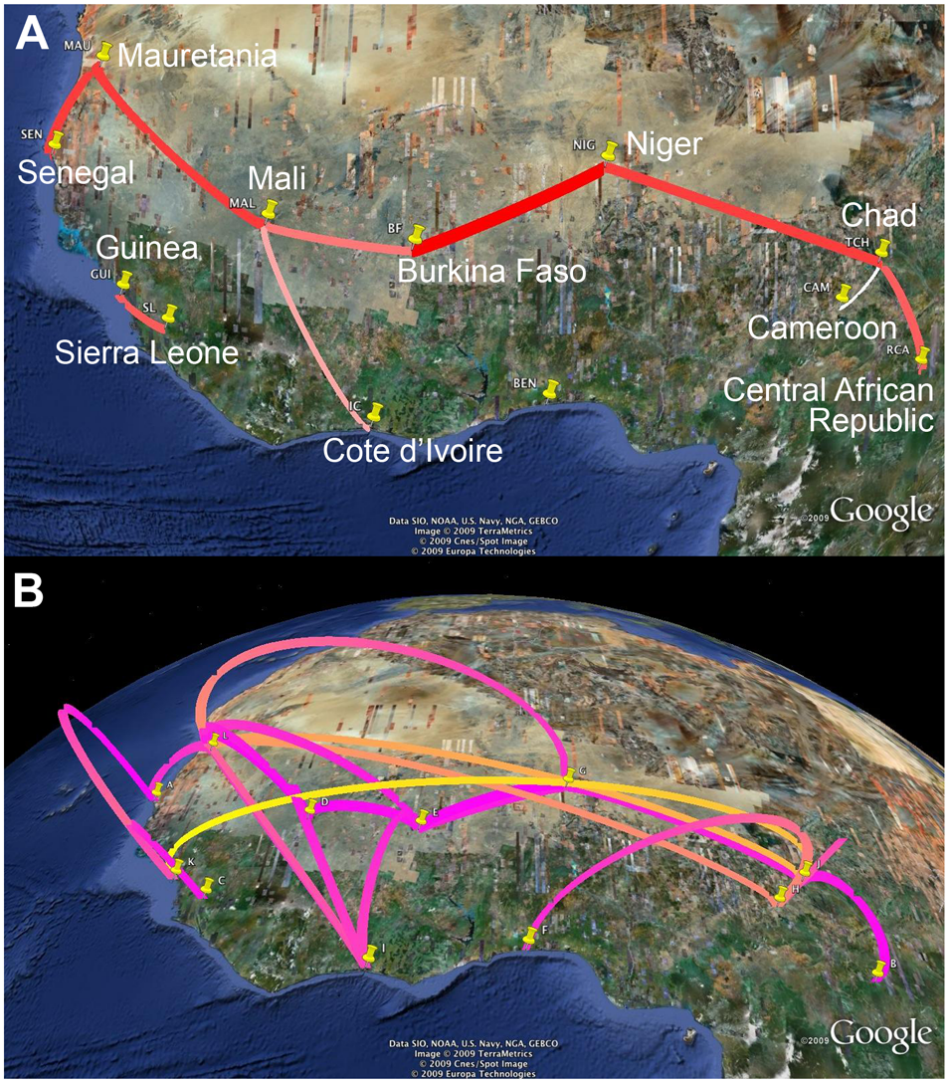
Inferred migration graph for African rabies virus and its reflection of the events reconstructed from the MCC tree. (A) Significantly non-zero migration rate using a Bayes factor test. Line thicknesses and the white-red color gradient relate to relative posterior migration rate expectations. (B) Projection of reconstructed migration events. Link heights indicate the relative durations of the branches upon which the inferred migration occurs, while the yellow-magenta color gradient informs the relative age of the transition (older-recent). The maps are based on satellite pictures made available in Google Earth (http://earth.google.com).

Although geographic origins remain elusive, we are able to identify locations that are epidemiologically linked using the BF test under BSSVS (Figure 7). Panel A in the figure highlights the rates yielding a BF
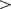
3. The resulting migration graph is markedly parsimonious with a distinctive East-West axis running along the Southern border of the Saharan desert. To glean how this graph reflects the migratory process acting along the rabies phylogeny, panel B projects each of the branches of the MCC phylogeny onto the geographic map. In this projection, we translate each branch into a geographic link that connects the branch's most probable starting and ending location states. The height of a link represents the relative length of the time elapsed on the link's corresponding branch, while the color gradient reflects the relative age of the migration. Many recent (magenta) migration events that occur in a relatively short time contribute to the well-supported East-West axis.

**Figure 8:**
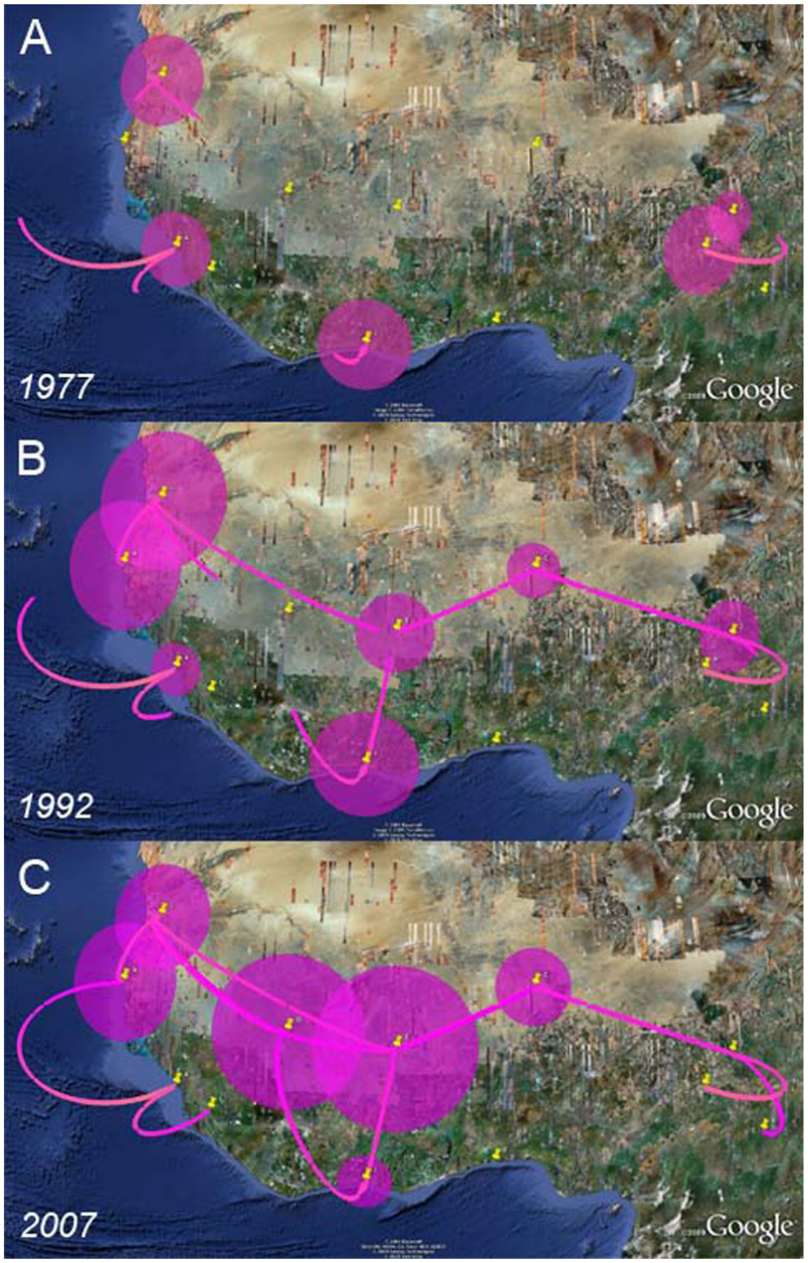
West African dog rabies virus migration over the last three decades. The different panels represent temporal projections of reconstructed migration events every 15 years: A) 1977, B) 1992 and C) 2007. In these projections, each MCC branch is again translated into a geographic link that connects the branch's most probable starting and ending location states. The panels only show migration events or partial migration events that have occurred up to a particular date, assuming that the virus migrates at a constant rate over the inferred time span of the branch. The maps are based on satellite pictures made available in Google Earth (http://earth.google.com).

Although the best supported rates mainly form an East-West axis, many transitions along this axis occur in the last three decades; this suggests that the axis is not representative of a relatively slow unidirectional migration wave. Figure 8 reports the migration pathways over the last thirty years. These migration events accumulating over time, contingent on the estimated time of the branches on which they occur, demonstrate that RABV diffusion in West Africa is characterized by different simultaneous migration events in various directions rather than a unidirectional pattern, and that most of these migrations are short-range, occurring between neighboring countries.

## Discussion

The Bayesian phylogeographic inference framework we present here incorporates the spatial and temporal dynamics of gene flow. In this study, we focus on pathogen diffusion because viral sequence sampling on a time-scale commensurate with the rate of substitution permits the inference of spatial patterns in real-time units. In addition, elucidating the phylodynamics of viral epidemics has important implications for public health management. We selected the Avian influenza A-H5N1 example to allow a convenient comparison of Bayesian ancestral state inference with the previous parsimony analysis; on the other hand, statistical analysis of the rabies migration in Africa up to this point has been largely unexplored. Both zoonoses represent a clear threat to human health. The frequent transmission of A-H5N1 from poultry or wild birds to humans suggest that the virus could emerge as or contribute genetically to the next human flu pandemic. Although the lack of a human-to-human transmission mechanism means that rabies will not emerge as a purely human disease, rabies infection causes a fatal neurological disease and at least 55,000 people die from this disease every year, mainly in the developing world [40].

A Bayesian statistical approach presents many advantages over parsimony inference of ancestral states. First, MCMC offers a computational technique to integrate over an unknown phylogeny and unknown migration process as the former is not directly observable in nature and the latter is poorly understood. Accommodating this lack of knowledge protects against potentially severe bias, but can reduce the power to make inferences; phylogeographic analyses are no exception to this. One can regard this uncertainty itself as a ‘mixed blessing’ because whilst it can hamper drawing definitive conclusions [13], it protect us from making overstated conclusions. For example, parsimony analysis of the influenza data establishes an epidemiological link between Guangdong and Indonesia [22]. Bayesian inference does not confirm this conclusion and phylogenetic analysis of more recently obtained sequence data now identifies the progenitors of Indonesian strains in the Chinese province of Hunan [26], a site which our shared analysis also identified as a possible source. Further, unlike parsimony, likelihood-based probabilistic methods consider branch lengths in ancestral reconstructions. The impact of the tree depth on root state reconstructions for the A-H5N1 genome segments clearly illustrates the importance of branch lengths. Moreover, probabilistic methods allow for estimating the relative posterior probability of each location state at any position along the phylogeny; this ability is indispensable in a hypothesis testing framework. As introduced by [19] in a different setting, we also demonstrate how phylogeographic parameters can be estimated from different genomic segments without assuming the same evolutionary history. H5N1 reassortment, however, will have not have fully unlinked HA and NA evolutionary histories and partially shared ancestry may lead to overstated credibility in some aspects of the phylogeographic inference.

Bayesian inference also proffers particular benefits within the class of likelihood-based methods, for example, by allowing for straightforward approaches to control model complexity. BSSVS naturally provides a BF test to identify significant non-zero migration rates. Further prior specification easily incorporates geographical detail of the sequence data. Although distance-informed priors appear to have little impact on the phylogeographic analyses presented here, both BSSVS and informed priors furnish new opportunities for hypothesis testing when comparing competing prior scenarios of the diffusion process. Examples include “gravity models” [41] in which infinitesimal rates become functions of the host population-sizes at the end-point locations and *a priori* structurally-fixed graphs [19]. Finally, it has been recognized that an MCMC-based Bayesian framework is well-suited to bring together information of different kinds [42]. The BEAST software, which has a strong focus on calibrated phylogenies and genealogies, elegantly illustrates this by offering a large number of complementary evolutionary models including substitution models, demographic and relaxed clock models that can be combined into a full probabilistic model [43]. By adding spatial reconstruction to this arsenal of evolutionary models, the full probabilistic inference now brings us much closer to biogeographical history reconstruction from genetic data.

Our primary motivation for exploiting BSSVS to select among all possible migration graphs is to elucidate the limited number of epidemiological links that appropriately explain the viral diffusion process. This parsimonious set both informs major modes of migration and reduces the high statistical variance that burdens estimation of all pairwise transition rates. Following this argument, less uncertainty on node state reconstructions would be expected when focusing on a parsimonious parameterization of the instantaneous rate matrix. The A-H5N1 analysis indeed indicates lower uncertainty of root state reconstructions. However, for some other internal nodes, we note the opposite behavior. We attribute this to the reversibility assumption in the rate matrix. Selection of reversible rates by BSSVS imposes more balanced transitions in the phylogeny among locations that could have unidirectional links in reality. Therefore, work is in progress to develop non-reversible models that may better fit a spatially expanding epidemic like A-H5N1 or recurring epidemic influenza emergence through source-sink dynamics [2]. Considerable technical hurdles remain to incorporate BSSVS procedures under such models. Because BSSVS places non-negligible probability on structural zeros in the rate matrix, we can not guarantee that all resulting rate matrices are diagonalizable, challenging stable computation. Bearing in mind the reversibility assumption, we pass no judgement on the origin of the A-H5N1 epidemic based on the frequency by which a location is present in well-supported rates, as was previously done in the parsimony analysis [22]. Instead, we focus on node location state reconstructions throughout the phylogeny and their posterior probabilities. Figure 1 suggests that, although Hong Kong and Guangdong both receive posterior support as root location states, the dominant location throughout the phylogeny and hence the more likely hub of diffusion is Guangdong. An inherent assumption of the discrete model of location change is that ancestral viruses necessarily reside at only the sampled locations of the extant viruses. In this respect, it is important to realize that the CTMC process should describe the underlying spatial dynamics more accurately as the sampling density increases. E.g., for A-H5N1 [26], provide more recently obtained sequence data across a larger set of geographic locations; the data could inform further pathways seeding remote localities that remain elusive in our present analysis. In addition to tackling the reversibility assumption, it may also prove necessary to relax constant diffusion rates through time to realistically model phylogeographic processes in many situations. Covarion-like models [44] and allowing different diffusion matrices across different time intervals in the phylogeny may help achieve these aims.

Our rabies phylogeographic analysis confirms a longstanding presence of this viral lineage in West Africa [28]. The virus appears to have a constant population size for about 150 years during which, extrapolating from the more recent spatial dynamics, diffusion occurs continuously with no particular directionality (Figure 6A). These continuous dynamics explain why we can not achieve precise root state location inference based only on samples from the last 20 years. In the light of the constant population dynamics, however, the location of the MRCA may be epidemiologically irrelevant as the location probably does not necessarily represent the ultimate source of the rabies endemic. We note that our analysis does not include all currently available strains originating from Chad, which may add to the weak East-to-West dispersal signal revealed by a recent parsimony analysis [39]. Our analysis confirms the model proposed for dog RABV in general; that is, of a series of spatially distinct clusters that experience relatively little contact among them [28],[39]. By providing a time scale for the seeding of these spatial clusters, we again demonstrate a clear advantage of the Bayesian inference over parsimony analysis. The ability to draw migrations over time also promotes a more precise dissection of local and temporal RABV movement on smaller geographical scales. After migration, the virus appears to establish local populations maintaining the viral lineage for at least a limited amount of time. These dynamics are reminiscent of a metapopulation model with continuous turnover of locally established viral populations. A long-standing rabies presence in West Africa is not surprising; already recognized since the late 60s, the territory plays a major role in the rabies-canid ecological balance [45]. It remains a remarkable feat that an acute and mainly fatal disease achieves prolonged endemicity. Because disease-induced mortality can rapidly deplete the number of susceptibles in a population, one expects epidemic cycles with oscillatory dynamics to occur. Rabies cycles and traveling waves have been well documented in wildlife across Europe and North America, e.g. [46], and more recently [47], demonstrate such cycles in African dogs. Because their periodicity is notably shorter than expected from epidemiological models, the authors argue that intervention responses also impact the epidemic cycles [47]. Importantly, there is also a remarkable phase synchrony in rabies outbreaks across southern and eastern Africa, most pronounced for distances up to 1,000 km [47]. For oscillating systems in particular, it is well known that dispersal can generate population synchrony [48]. Because previous studies illustrate that even limited amounts of relatively local dispersal can generate synchrony in cyclical dynamics over large spatial scales [48], and that the resulting synchrony tends to decline as distance increases and varies through time [49], [47] argue that dispersal could enforce synchrony in dog rabies epidemics across different countries. Our analysis clearly reveals rabies dispersal as a continuous dynamic process that could indeed be essential in maintaining epidemic cycles. As argued by [39], however, the rate of dispersal is probably not sufficiently high to explain the short epidemic cycles as suggested by [47]. Nevertheless, we underscore that sustained and coordinated responses across political boundaries are necessary to control domestic dog rabies in Africa.

Many questions in evolutionary biology require a biogeographical perspective on the population under investigation. We hope to have demonstrated that Bayesian phylogeographic framework can contribute significantly to evolutionary hypothesis testing, and, although we have focused on viral phylodynamics, this approach is generally applicable in molecular evolution. Employing geographically-informed priors delivers a first step in incorporating GIS information. Future developments like irreversible CTMC processes may offer even more biological realism.

## Methods

For many spatial scales and problems, geography can naturally be partitioned into a finite number of discrete sites 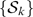 for 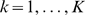. Examples of these situations include individual cities, islands or countries. Starting from the observed data, at the tips of the phylogeny 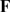 we record discretized locations 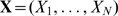, where 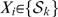 pin-points the sampling site of taxon 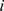. Unobserved in the spatial process are the locations of the most common recent ancestor 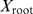 drawing from root distribution 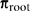, the times at which the descendent taxa move and amongst which discrete sites these moves occur, a process which ultimately gives rise to 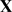. Conditioning on 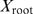 and the unobserved locations realized at each internal node 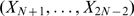 [13],[16], suggest modeling the instantaneous locations 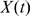 for taxa along each branch in 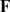 as independent continuous-time Markov chains (CTMCs). CTMCs are the same processes one commonly exploits to model sequence character evolution [15],[50]. Although many readers are familiar with CTMCs, we here highlight several chain properties to which we turn later when discussing CTMC modeling limitations. CTMCs are the simplest stochastic processes that emit discrete outcomes as a continuous function of time. The processes are memoryless, in that the probability of transitioning to a new location only depends on the current location and not the past history. A 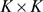 infinitesimal rate matrix 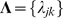 completely characterizes the CTMC process. Rate matrix 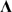 contains non-negative off-diagonal entries and all rows sum to 0, yielding a stochastic matrix upon exponentiation. Solving the Chapman-Kolmogorov equation that specifies the behavior of the chain yields the finite-time transition probabilities 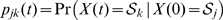. In matrix form,
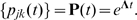
(1)Determining the finite-time transition probabilities involves matrix exponentiation, generally accomplished through an eigen-decomposition of 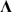. Here, we restrict ourselves to infinitesimal rate matrices that yield only real eigen-values and eigen-vectors. Any matrix similar to a symmetric matrix ensures a real eigen-decomposition; consequentially, we formulate
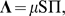
(2)where 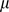 is an overall rate scalar, 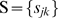 is a 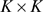 symmetric matrix and 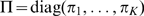. Infinitesimal rate matrices of this form generate reversible Markov chains, such that
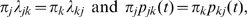
(3)placing many restrictions on the underlying geographic process. We discuss these limitations and modeling extensions that allow for irreversible chains in the Discussion. In its most general time-reversibile (GTR) form, 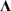 contains 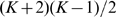 free parameters, with 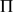 donating 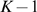 together with 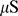
's 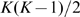 off-diagonal entries. Following standard practice, we normalize entries in 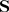 such that 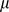 measures the expected (with respect to 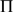) number of transitions per unit time 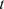.

One illuminating perspective from which to view a CTMC is that of a random walk on a graph 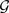. From this perspective, the possible realizations of the chain 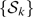 correspond to the vertex set of 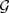. Between the vertices lie edges that record the infinitesimal transition rates. For example, between 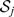 and 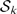 sits 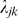. As a continuous-time random walk, a particle, starting at vertex 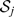 at time 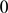, first waits an Exponential amount of time with rate 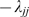 and then randomly decides to which neighboring vertix 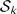 to move with probability 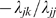. Now on 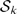, the process repeats. Neighboring vertices are those for which a single edge connects them. For character evolution, “complete” graphs find almost exclusive use, such that edges exist between all pairs of vertices. At a minimum, however, the graph must remain “connected”, such that it remains possible to walk between any two vertices on 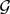.

### 

#### Bayesian stochastic search variable selection

When GTR models find use modeling nucleotide substitution, most of the 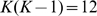 possible transitions have non-neglible probability of occurring and are observed over the evolutionary history. Such is unlikely to be the case for geographical locations; given that there may be many sites and each taxon only has one location (the equivalent of just one single alignment site), we expect most transitions to rarely, if ever, occur. Consequentially, we suspect *a priori* that many infinitesimal rates are zero. From a statistical perspective, so many degrees of freedom fit to the limited data lead to extremely high variance estimates. These poor estimates arise not only for 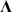, but, more critically, for inference of the unobserved ancestral locations and 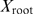. We circumvent this sparse data problem by invoking BSSVS to select a parsimonious parameterization of 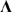. BSSVS enables us to simultaneously determine which infinitesimal rates are zero depending on the evidence in the data and efficiently infer the ancestral locations. As a beneficial by-product of BSSVS, directly quantifying the evidence about which rates are non-zero furnishes both the most likely migration patterns and the ability to test between competing migratory hypotheses.

BSSVS is traditionally applied to model selection problems in a linear regression framework, in which statisticians start with a large number of potential predictors 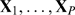 and ask which among these associate linearly with an 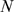-dimensional outcome variable 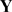. For example, the full model becomes 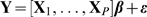, where 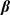 is a 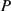-dimensional vector of regression coefficients and 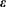 is an 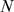-dimensional vector of normally distributed errors with mean 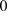. When 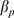 for 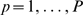 differs significantly from 0, 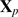 helps predict 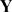, otherwise 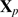 contributes little additional information and warrants removal from the model via forcing 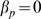. Given potentially high correlation between the predictors, deterministic model search strategies tend not to find the optimal set of predictors unless one explores all possible subsets. This exploration is generally computationally impractical as there exist 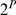 such subsets and completely fails for 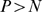.

Recent work in BSSVS [51],[52] efficiently performs the exploration in two steps. In the first step, the approach augments the model state-space with a set of 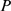 binary indicator variables 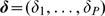 and imposes a prior 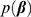 on the regression coefficients that has expectation 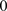 and variance proportional to a 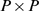 diagonal matrix with its entries equal to 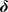. If 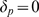, then the prior variance on 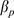 shrinks to 0 and enforces 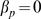 in the posterior. In the second step, MCMC explores the joint space of 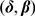 simultaneously.

To map BSSVS into the phylogeography setting, we consider selection among the 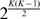 random graphs in which each of the 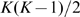 edges either exists or does not exist in 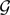. Let 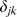 be the binary indicator that an edge exists connecting 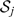 and 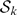. An equivalent parameterization specifies that 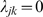 when 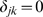 and 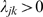 otherwise. So, rate matrix 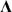 plays an analogous role to the regression coefficients in BSSVS. An important difference is that 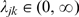 while 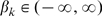, mandating alternative prior formulations.

#### Prior specification

To specify a prior distribution over 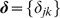, we assume that each indicator acts *a priori* as an independent Bernoulli random variable (RV) with small success probability 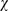. The sum of independent Bernoulli RVs yields a Binomial distribution over their sum 
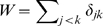
. In the limit that 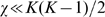, this prior conveniently collapses to
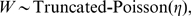
(4)where 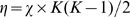 is the prior expected number of edges in graph 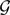.

We entertain two prior choices for 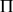. Diagonal vector 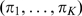 is the stationary distribution for the CTMC when all edges are included in the graph 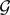. For this complete graph, as the length of the random walk 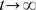, one expects that 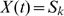 with probability 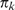. One natural choice says that there exists no spatial preference over the long-run and fixes 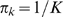 for all 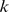. However, sites may expound spatial preference over the long-run; for example, such preference can relate to known or unknown quantities such as population-size or geographic size of the site. In these situations, we estimate 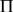 simultaneously with the rest of the model by imposing the flat prior 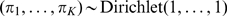. Also non-informatively for small values, we take 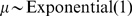.

To complete the CTMC specification, we assume that all unnormalized rates in 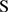 are *a priori* independent and Gamma-distributed with prior expectation 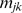 and variance 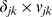, following in the vein of Bayesian SSVS. However, little previous work on prior formulation helps inform our choices of 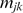 and 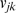. This represents a critically important area of research. A common, yet arbitrary choice in the Bayesian phylogenetic literature assumes that rates draw from Exponential distributions, forcing 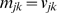. We follow this practice in light of there being no obvious way to elicit information on the variance of these rates. Finally, we explore two choices for setting the means. The first assumes no preference over rates, setting all 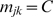, where 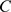 is an arbitrary constant; as, after normalization, only ratios of infinitesimal rates participate in the data likelihood, the actual value of 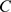 has no influence on the likelihood. The second is informed by the geographical distance between sites.

#### Distance informed prior

Considerable additional information exists about the sites 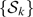 and remains unused. Most notably, the geographic distances 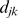 between (the centroids) of sites is readily measurable. *A priori* we may believe that more distantly separated sites have the smallest infinitesimal migration rates, yielding
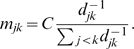
(5)Other information is also surely helpful and application-specific. One example involving human hosts quantifies the availability of motorized transportation, such as air flights, between sites. We explore the utility BSSVS and distance-informed priors in our phylogeographic models.

#### Bayes factor test of significant diffusion rates

The Bayes factor (BF) for a particular rate 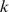 contributing to the migration graph is the posterior odds that rate 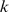 is non-zero divided by the equivalent prior odds,
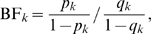
(6)where 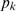 is the posterior probability that rate 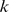 is non-zero, in this case the posterior expectation of indicator 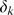. Since we employ a truncated Poisson prior with mean 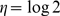, that assigns 50% prior probability on the minimal rate configuration (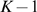), the prior probability 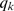 reduces to
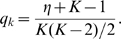
(7)We consider rates yielding a BF
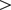
3 as well supported diffusion rates constituting the migration graph.

### Sequence data, joint modeling and posterior sampling

A strength of the Bayesian approach we exploit in this paper is the ability to integrate together into a joint model of spatial locations 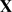 and aligned molecular sequence data 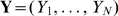 collected from the 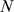 taxa. The joint model affords a natural way to incorporate uncertainty about the unobserved phylogeny 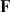 and the character substitution process giving rise to 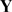. We take a standard statistical phylogenetic approach and assume that a separate CTMC characterized by 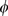 generates 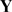. While we discuss specific choices about this process in the Results sections, we do assume that the sequence and location CTMCs are independent given 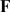, enabling us to write the joint model posterior distribution as
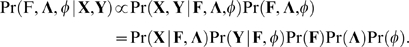
(8)Likelihoods 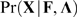 and 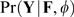 follow directly from Felsenstein's pruning algorithm [15], efficiently integrating over all possible locations and sequences at the root and internal nodes in 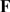.

We approximate the joint posterior (8) and its marginalizations using MCMC implemented in the software package BEAST [43]. We employ standard transition kernels over the parameter spaces of 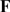 and 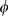. To sample realizations of 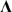, we consider random-walk operators on the continuous portions and a specialized “bit-flip” operator on the Bernoulli rate indicators 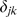. [53] discuss this transition kernel further. Finally, in many situations, inference on the posterior distribution of the root and internal node states is of paramount interest. We implement a pre-order, tree-traversal algorithm in BEAST that allows researchers to generate realizations of the root and internal node states following [20] and produce posterior summaries. Importantly, this procedure is not limited to phylogeographic models, making general ancestral state reconstruction available in BEAST for the first time.

#### Summarizing posterior location uncertainty

An important statistical question asks to what extent the data inform our inference when fitting different phylogeographic models. A model of low statistical power makes poor use of the information in the data, while a successful model exploits this information to generate posterior distributions that are maximally different from prior beliefs. One primary outcome of a Bayesian phylogeographic study is the marginal posterior distribution of the root location 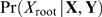. We calculate the Kullback-Leibler (KL) divergence [54] from the root location prior 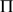 to summarize this information gain,
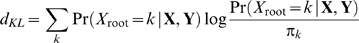
(9)where 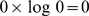. When the posterior and prior distributions are equal, 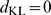. In the examples in this paper, we fix 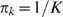 and 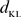 achieves its maximum 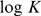 when the posterior places all estimable mass on a single location. From this perspective, 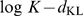 plays the role of a measure of dispersion [55] or uncertainty. As a simple numerical summary, we also use 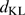 to explore the utility of BSSVS and distance-informed priors on drawing inference from phylogeographic models. Larger values signify that the model extracts more information from the data. To calculate KL divergence, we employ a uniform discrete distribution as reference distribution.

#### Association index

Following existing phylogeographic approaches, we finally score the degree of spatial admixture using a modified association index (AI) [35]. For a given phylogeny 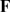 and tip locations 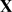, we obtain the association value 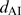 by summing over each internal node 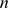,
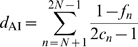
(10)where 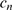 counts the number of sampled locations descendent to 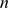 and 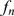 is the frequency of the highest frequency location amongst these descendents. Similar to [10], we report the posterior distributions 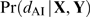 and the AI compares these distributions to those obtained by random permutation of the tip locations 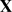. Deviation from these permuted distributions reflected in low AI values suggests phylogeographic structure whereas AI values close to 1 suggest spatial admixture.

#### Visualizing phylogeographic diffusion

To summarize the posterior distribution of ancestral location states, we annotate nodes in the MCC tree with the modal location state for each node using TreeAnnotator, and visualize this tree using FigTree (available at http://tree.bio.ed.ac.uk/software). To provide a spatial projection, we convert the tree into a keyhole markup language (KML) file suitable for viewing with Google Earth (http://earth.google.com). We introduce the temporal information on the marked-up tree using the 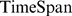 KML-function to animate viral dispersal over the time. Example KML files for the Avian Influenza A HA and NA genes are included as supplementary files and software to convert annotated trees to KML is available from the authors on request.

## Supporting Information

Dataset S1 KML file for H5N1 diffusion over time as inferred from HA
(2.13 MB XML)

Dataset S2 Supplementary information: KML file for H5N1 diffusion over time as inferred from NA
(2.10 MB XML)

## References

[1] Holmes E (2004). The phylogeography of human viruses.. Molecular Ecology.

[2] Rambaut A, Pybus O, Nelson M, Viboud C, Taubennerger J (2008). The genomic and epidemiological dynamics of human influenza A virus.. Nature.

[3] Russell CA, Jones TC, Barr IG, Cox NJ, Garten RJ (2008). The global circulation of seasonal influenza A (H3N2) viruses.. Science.

[4] Wallace RG, Fitch WM (2008). Influenza A H5N1 immigration is filtered out at some international borders.. PLoS ONE.

[5] Olsen SJ, Chang HL, Cheung TYY, Tang AFY, Fisk TL (2003). Transmission of the severe acute respiratory syndrome on aircraft.. N Engl J Med.

[6] Knowles L, Maddison W (2002). Statistical phylogeography.. Molecular Ecology.

[7] Slatkin M, Maddison WP (1989). A cladistic measure of gene flow inferred from the phylogenies of alleles.. Genetics.

[8] Templeton A (2004). Statistical phylogeography: methods for evaluating and minimizing inference errors.. Molecular Ecology.

[9] Lanciotti R, Gubler D, Trent D (1997). Molecular evolution and phylogeny of dengue-4 viruses.. Journal of General Virology.

[10] Parker J, Rambaut A, Pybus OG (2008). Correlating viral phenotypes with phylogeny: accounting for phylogenetic uncertainty.. Infect Genet Evol.

[11] Zarate S, Pond SLK, Shapshak P, Frost SDW (2007). Comparative study of methods for detecting sequence compartmentalization in human immunodeficiency virus type 1.. J Virol.

[12] D Swofford WM (1992).

[13] Ronquist F (2004). Bayesian inference of character evolution.. Trends in Ecology and Evolution.

[14] Cunningham C, Omland K, Oakley T (1998). Reconstructing ancestral character states: a critical reappraisal.. Trends in Ecology & Evolution.

[15] Felsenstein J (1981). Evolutionary trees from DNA sequences: a maximum likelihood approach.. Journal of Molecular Evolution.

[16] Pagel M (1999). The maximum likelihood approach to reconstructing ancestral character states of discrete characters on phylogenies.. Systematic Biology.

[17] Minin V, Suchard M (2008). Fast, accurate and simulation-free stochastic mapping.. Philosophical Transactions of the Royal Society B: Biological Sciences.

[18] Liò P, Goldman N (1998). Models of molecular evolution and phylogeny.. Genome Research.

[19] Sanmartin I, van der Mark P, Ronquist F (2008). Inferring disperal: a Bayesian approach to phylogeny-based island biogeography, with special reference to the Canary Islands.. Journal of Biogeography.

[20] Pagel M, Meade A, Barker D (2004). Bayesian estimation of ancestral character states on phylogenies.. Systematic Biology.

[21] Pond SLK, Frost SDW, Muse SV (2005). Hyphy: hypothesis testing using phylogenies.. Bioinformatics.

[22] Wallace R, HoDac H, Lathrop R, Fitch W (2007). A statistical phylogeography of influenza A H5N1.. Proceedings of the National Academy of Sciences, USA.

[23] Pybus O, Rambaut A, Harvey P (2000). An integrated framework for the inference of viral population history from reconstructed genealogies.. Genetics.

[24] Drummond A, Pybus O, Rambaut A, Forsberg R, Rodrigo A (2003). Measurably evolving populations.. Trends in Ecology & Evolution.

[25] Drummond A, Rambaut A, Shapiro B, Pybus O (2005). Bayesian coalescent inference of past population dynamics from molecular sequences.. Molecular Biology and Evolution.

[26] Wang J, Vijaykrishna D, Duan L, Bahl J, Zhang JX (2008). Identification of the progenitors of Indonesian and Vietnamese avian influenza A (H5N1) viruses from southern China.. J Virol.

[27] Bourhy H, Kissi B, Tordo N (1993). Molecular diversity of the lyssavirus genus.. Virology.

[28] Bourhy H, Reynes JM, Dunham EJ, Dacheux L, Larrous F (2008). The origin and phylogeography of dog rabies virus.. J Gen Virol.

[29] Hasegawa M, Kishino H, Yano T (1985). Dating the human-ape splitting by a molecular clock of mitochondrial DNA.. Journal of Molecular Evolution.

[30] Yang Z (1995). A space-time process model for the evolution of DNA sequences.. Genetics.

[31] Kingman J (2000). Origins of the coalescent: 1974–1982.. Genetics.

[32] Vijaykrishna D, Bahl J, Riley S, Duan L, Zhang JX (2008). Evolutionary dynamics and emergence of panzootic H5N1 influenza viruses.. PLoS Pathog.

[33] Xu X, Cox NJ, Guo Y (1999). Genetic characterization of the pathogenic influenza A/Goose/Guangdong/1/96 (H5N1) virus: similarity of its hemagglutinin gene to those of H5N1 viruses from the 1997 outbreaks in Hong Kong.. Virology.

[34] Subbarao K, Klimov A, Katz J, Regnery H, Lim W (1998). Characterization of an avian influenza A (H5N1) virus isolated from a child with a fatal respiratory illness.. Science.

[35] Wang TH, Donaldson YK, Brettle RP, Bell JE, Simmonds P (2001). Identification of shared populations of human immunodeficiency virus type 1 infecting microglia and tissue macrophages outside the central nervous system.. J Virol.

[36] Kilpatrick AM, Chmura AA, Gibbons DW, Fleischer RC, Marra PP (2006). Predicting the global spread of H5N1 avian influenza.. Proc Natl Acad Sci U S A.

[37] Chen H, Li Y, Li Z, Shi J, Shinya K (2006). Properties and dissemination of H5N1 viruses isolated during an influenza outbreak in migratory waterfowl in western China.. J Virol.

[38] Davis PL, Rambaut A, Bourhy H, Holmes EC (2007). The evolutionary dynamics of canid and mongoose rabies virus in Southern Africa.. Arch Virol.

[39] Talbi C, Holmes EC, de Benedictis P, Faye O, Nakouné E (2009). Evolutionary history and dynamics of dog rabies virus in western and Central Africa.. J Gen Virol.

[40] Knobel DL, Cleaveland S, Coleman PG, Fevre EM, Meltzer MI (2005). Re-evaluating the burden of rabies in Africa and Asia.. Bull World Health Organ.

[41] Viboud C, Bjornstad O, Smith D, Simonsen L, Miller M (2006). Synchrony, waves and spatial hierarchies in the spread of influenza.. Science.

[42] Drummond A, Nicholls G, Rodrigo A, Solomon W (2002). Estimating mutation parameters, population history and genealogy simultaneously from temporally spaced sequence data.. Genetics.

[43] Drummond AJ, Rambaut A (2007). Beast: Bayesian evolutionary analysis by sampling trees.. BMC Evol Biol.

[44] Fitch WM, Markowitz E (1970). An improved method for determining codon variability in a gene and its application to the rate of fixation of mutations in evolution.. Biochem Genet.

[45] Chalmers A, Scott G (1969). Ecology of rabies.. Tropical Animal Health and Production.

[46] Anderson R, Jackson H, May R, Smith A (1981). Population dynamics of fox rabies in Europe.. Nature.

[47] Hampson K, Dushoff J, Bingham J, Bruckner G, Ali YH (2007). Synchronous cycles of domestic dog rabies in sub-Saharan Africa and the impact of control efforts.. Proc Natl Acad Sci U S A.

[48] Bjornstad O, Ims R, Lambin X (1999). Spatial population dynamics: analyzing patterns and processes of population synchrony.. Trends Ecol Evol.

[49] Ranta E, Kaitala V, Lundberg P (1998). Population variability in space and time: the dynamics of synchronous population fluctuations.. Oikos.

[50] Jukes T, Cantor C, Munro H (1969). Evolution of protein molecules.. Mammaliam Protein Metabolism.

[51] Kuo L, Mallick B (1998). Variable selection for regression models.. Sankhya B.

[52] Chipman H, George E, McCulloch R (2001). The practical implementation of Bayesian model selection.. IMS Lecture Notes – Monograph Series.

[53] Drummond A, Suchard M (In submission). Bayesian random local clocks, or one rate to rule them all.. Systematic Biology?.

[54] Kullback S, Leibler R (1951). On information and sufficiency.. Annals of Mathematical Statistics.

[55] Gilulua Z, Haberman S (1995). Dispersion of categorical variables and penalty functions: derivation, estimation and comparability.. Journal of the American Statistical Association.

